# Supplementary material for: Sex-Specific Cut-Offs of Single Point Insulin Sensitivity Estimator (SPISE) in Predicting Metabolic Syndrome in the Arab Adolescents
Source: Diagnostics (Basel). 2023 Jan 16;13(2):324. doi: 10.3390/diagnostics13020324 (PMC9858553; doi:10.3390/diagnostics13020324)
Supplement: Supplementary file 1 [file diagnostics-13-00324-s001.zip › diagnostics-2124512-supplementary.docx]

**Table S1:** ROC-SPISE coordinates for All subjects

| Criterion | Sensitivity | 95% CI | Specificity | 95% CI | +LR | -LR |
| --- | --- | --- | --- | --- | --- | --- |
| <2.03 | 0.00 | 0.0 - 4.5 | 100.00 | 99.5 - 100.0 |  | 1.00 |
| ≤2.15 | 2.47 | 0.3 - 8.6 | 100.00 | 99.5 - 100.0 |  | 0.98 |
| ≤2.44 | 2.47 | 0.3 - 8.6 | 99.87 | 99.3 - 100.0 | 19.28 | 0.98 |
| ≤2.95 | 12.35 | 6.1 - 21.5 | 99.87 | 99.3 - 100.0 | 96.42 | 0.88 |
| ≤2.97 | 12.35 | 6.1 - 21.5 | 99.74 | 99.1 - 100.0 | 48.21 | 0.88 |
| ≤3.24 | 17.28 | 9.8 - 27.3 | 99.74 | 99.1 - 100.0 | 67.49 | 0.83 |
| ≤3.4 | 17.28 | 9.8 - 27.3 | 99.49 | 98.7 - 99.9 | 33.75 | 0.83 |
| ≤3.49 | 19.75 | 11.7 - 30.1 | 99.49 | 98.7 - 99.9 | 38.57 | 0.81 |
| ≤3.51 | 19.75 | 11.7 - 30.1 | 99.23 | 98.3 - 99.7 | 25.71 | 0.81 |
| ≤3.64 | 23.46 | 14.8 - 34.2 | 99.23 | 98.3 - 99.7 | 30.53 | 0.77 |
| ≤3.65 | 23.46 | 14.8 - 34.2 | 99.10 | 98.2 - 99.6 | 26.17 | 0.77 |
| ≤3.68 | 25.93 | 16.8 - 36.9 | 99.10 | 98.2 - 99.6 | 28.93 | 0.75 |
| ≤3.69 | 28.40 | 18.9 - 39.5 | 98.98 | 98.0 - 99.6 | 27.72 | 0.72 |
| ≤3.71 | 29.63 | 20.0 - 40.8 | 98.98 | 98.0 - 99.6 | 28.93 | 0.71 |
| ≤3.74 | 29.63 | 20.0 - 40.8 | 98.59 | 97.5 - 99.3 | 21.04 | 0.71 |
| ≤3.76 | 30.86 | 21.1 - 42.1 | 98.46 | 97.3 - 99.2 | 20.09 | 0.70 |
| ≤3.8 | 33.33 | 23.2 - 44.7 | 98.46 | 97.3 - 99.2 | 21.69 | 0.68 |
| ≤3.81 | 33.33 | 23.2 - 44.7 | 98.34 | 97.2 - 99.1 | 20.03 | 0.68 |
| ≤3.85 | 37.04 | 26.6 - 48.5 | 98.34 | 97.2 - 99.1 | 22.25 | 0.64 |
| ≤3.88 | 37.04 | 26.6 - 48.5 | 98.21 | 97.0 - 99.0 | 20.66 | 0.64 |
| ≤3.92 | 38.27 | 27.7 - 49.7 | 98.21 | 97.0 - 99.0 | 21.35 | 0.63 |
| ≤4 | 38.27 | 27.7 - 49.7 | 97.82 | 96.5 - 98.7 | 17.58 | 0.63 |
| ≤4.01 | 39.51 | 28.8 - 51.0 | 97.82 | 96.5 - 98.7 | 18.15 | 0.62 |
| ≤4.16 | 39.51 | 28.8 - 51.0 | 97.70 | 96.4 - 98.6 | 17.14 | 0.62 |
| ≤4.18 | 40.74 | 29.9 - 52.2 | 97.44 | 96.1 - 98.4 | 15.91 | 0.61 |
| ≤4.19 | 41.98 | 31.1 - 53.5 | 97.31 | 95.9 - 98.3 | 15.61 | 0.60 |
| ≤4.21 | 43.21 | 32.2 - 54.7 | 97.06 | 95.6 - 98.1 | 14.67 | 0.59 |
| ≤4.37 | 43.21 | 32.2 - 54.7 | 96.03 | 94.4 - 97.3 | 10.89 | 0.59 |
| ≤4.39 | 44.44 | 33.4 - 55.9 | 96.03 | 94.4 - 97.3 | 11.20 | 0.58 |
| ≤4.43 | 44.44 | 33.4 - 55.9 | 95.13 | 93.4 - 96.5 | 9.13 | 0.58 |
| ≤4.46 | 45.68 | 34.6 - 57.1 | 95.13 | 93.4 - 96.5 | 9.39 | 0.57 |
| ≤4.51 | 45.68 | 34.6 - 57.1 | 94.62 | 92.8 - 96.1 | 8.49 | 0.57 |
| ≤4.54 | 46.91 | 35.7 - 58.3 | 94.37 | 92.5 - 95.9 | 8.33 | 0.56 |
| ≤4.55 | 46.91 | 35.7 - 58.3 | 94.24 | 92.4 - 95.8 | 8.14 | 0.56 |
| ≤4.56 | 48.15 | 36.9 - 59.5 | 94.11 | 92.2 - 95.7 | 8.17 | 0.55 |
| ≤4.57 | 50.62 | 39.3 - 61.9 | 93.98 | 92.1 - 95.5 | 8.41 | 0.53 |
| ≤4.6 | 50.62 | 39.3 - 61.9 | 93.73 | 91.8 - 95.3 | 8.07 | 0.53 |
| ≤4.62 | 51.85 | 40.5 - 63.1 | 93.60 | 91.6 - 95.2 | 8.10 | 0.51 |
| ≤4.68 | 51.85 | 40.5 - 63.1 | 92.96 | 90.9 - 94.7 | 7.36 | 0.52 |
| ≤4.69 | 53.09 | 41.7 - 64.3 | 92.96 | 90.9 - 94.7 | 7.54 | 0.50 |
| ≤4.73 | 53.09 | 41.7 - 64.3 | 92.32 | 90.2 - 94.1 | 6.91 | 0.51 |
| ≤4.74 | 54.32 | 42.9 - 65.4 | 92.32 | 90.2 - 94.1 | 7.07 | 0.49 |
| ≤4.78 | 54.32 | 42.9 - 65.4 | 92.06 | 89.9 - 93.9 | 6.84 | 0.50 |
| ≤4.83 | 55.56 | 44.1 - 66.6 | 91.93 | 89.8 - 93.7 | 6.89 | 0.48 |
| ≤4.89 | 55.56 | 44.1 - 66.6 | 91.29 | 89.1 - 93.2 | 6.38 | 0.49 |
| ≤4.9 | 56.79 | 45.3 - 67.8 | 91.29 | 89.1 - 93.2 | 6.52 | 0.47 |
| ≤4.99 | 56.79 | 45.3 - 67.8 | 90.52 | 88.3 - 92.5 | 5.99 | 0.48 |
| ≤5.03 | 58.02 | 46.5 - 68.9 | 90.52 | 88.3 - 92.5 | 6.12 | 0.46 |
| ≤5.12 | 58.02 | 46.5 - 68.9 | 89.63 | 87.3 - 91.7 | 5.59 | 0.47 |
| ≤5.14 | 59.26 | 47.8 - 70.1 | 89.63 | 87.3 - 91.7 | 5.71 | 0.45 |
| ≤5.23 | 59.26 | 47.8 - 70.1 | 87.84 | 85.3 - 90.0 | 4.87 | 0.46 |
| ≤5.26 | 60.49 | 49.0 - 71.2 | 87.84 | 85.3 - 90.0 | 4.97 | 0.45 |
| ≤5.28 | 60.49 | 49.0 - 71.2 | 87.58 | 85.1 - 89.8 | 4.87 | 0.45 |
| ≤5.31 | 61.73 | 50.3 - 72.3 | 87.58 | 85.1 - 89.8 | 4.97 | 0.44 |
| ≤5.33 | 61.73 | 50.3 - 72.3 | 87.45 | 84.9 - 89.7 | 4.92 | 0.44 |
| ≤5.36 | 62.96 | 51.5 - 73.4 | 87.32 | 84.8 - 89.6 | 4.97 | 0.42 |
| ≤5.38 | 62.96 | 51.5 - 73.4 | 87.07 | 84.5 - 89.3 | 4.87 | 0.43 |
| ≤5.39 | 64.20 | 52.8 - 74.6 | 86.94 | 84.4 - 89.2 | 4.92 | 0.41 |
| ≤5.44 | 64.20 | 52.8 - 74.6 | 86.68 | 84.1 - 89.0 | 4.82 | 0.41 |
| ≤5.45 | 65.43 | 54.0 - 75.7 | 86.68 | 84.1 - 89.0 | 4.91 | 0.40 |
| ≤5.51 | 65.43 | 54.0 - 75.7 | 86.17 | 83.5 - 88.5 | 4.73 | 0.40 |
| ≤5.52 | 66.67 | 55.3 - 76.8 | 86.04 | 83.4 - 88.4 | 4.78 | 0.39 |
| ≤5.53 | 67.90 | 56.6 - 77.8 | 85.92 | 83.3 - 88.3 | 4.82 | 0.37 |
| ≤5.54 | 67.90 | 56.6 - 77.8 | 85.79 | 83.1 - 88.2 | 4.78 | 0.37 |
| ≤5.56 | 69.14 | 57.9 - 78.9 | 85.66 | 83.0 - 88.0 | 4.82 | 0.36 |
| ≤5.65 | 69.14 | 57.9 - 78.9 | 84.64 | 81.9 - 87.1 | 4.50 | 0.36 |
| ≤5.69 | 70.37 | 59.2 - 80.0 | 84.51 | 81.8 - 87.0 | 4.54 | 0.35 |
| ≤5.8 | 70.37 | 59.2 - 80.0 | 83.48 | 80.7 - 86.0 | 4.26 | 0.35 |
| ≤5.82 | 71.60 | 60.5 - 81.1 | 83.48 | 80.7 - 86.0 | 4.34 | 0.34 |
| ≤5.9 | 71.60 | 60.5 - 81.1 | 82.59 | 79.7 - 85.2 | 4.11 | 0.34 |
| ≤5.91 | 72.84 | 61.8 - 82.1 | 82.59 | 79.7 - 85.2 | 4.18 | 0.33 |
| ≤5.94 | 72.84 | 61.8 - 82.1 | 82.20 | 79.3 - 84.8 | 4.09 | 0.33 |
| ≤5.98 | 74.07 | 63.1 - 83.2 | 82.20 | 79.3 - 84.8 | 4.16 | 0.32 |
| ≤6.13 | 74.07 | 63.1 - 83.2 | 80.67 | 77.7 - 83.4 | 3.83 | 0.32 |
| ≤6.14 | 75.31 | 64.5 - 84.2 | 80.67 | 77.7 - 83.4 | 3.90 | 0.31 |
| ≤6.23 | 75.31 | 64.5 - 84.2 | 79.26 | 76.2 - 82.1 | 3.63 | 0.31 |
| ≤6.24 | 77.78 | 67.2 - 86.3 | 79.13 | 76.1 - 81.9 | 3.73 | 0.28 |
| ≤6.31 | 77.78 | 67.2 - 86.3 | 78.36 | 75.3 - 81.2 | 3.59 | 0.28 |
| ≤6.33 | 79.01 | 68.5 - 87.3 | 78.36 | 75.3 - 81.2 | 3.65 | 0.27 |
| ≤6.35 | 79.01 | 68.5 - 87.3 | 77.98 | 74.9 - 80.8 | 3.59 | 0.27 |
| ≤6.37 | 80.25 | 69.9 - 88.3 | 77.98 | 74.9 - 80.8 | 3.64 | 0.25 |
| ≤6.45 | 80.25 | 69.9 - 88.3 | 76.82 | 73.7 - 79.7 | 3.46 | 0.26 |
| **≤6.46** | **81.48** | **71.3 - 89.2** | **76.82** | **73.7 - 79.7** | **3.52** | **0.24** |
| ≤6.61 | 81.48 | 71.3 - 89.2 | 75.54 | 72.4 - 78.5 | 3.33 | 0.25 |
| ≤6.62 | 82.72 | 72.7 - 90.2 | 75.42 | 72.2 - 78.4 | 3.36 | 0.23 |
| ≤6.92 | 82.72 | 72.7 - 90.2 | 71.19 | 67.9 - 74.3 | 2.87 | 0.24 |
| ≤6.93 | 83.95 | 74.1 - 91.2 | 71.19 | 67.9 - 74.3 | 2.91 | 0.23 |
| ≤7.08 | 83.95 | 74.1 - 91.2 | 69.78 | 66.4 - 73.0 | 2.78 | 0.23 |
| ≤7.09 | 85.19 | 75.6 - 92.1 | 69.78 | 66.4 - 73.0 | 2.82 | 0.21 |
| ≤7.1 | 86.42 | 77.0 - 93.0 | 69.53 | 66.2 - 72.7 | 2.84 | 0.20 |
| ≤7.2 | 86.42 | 77.0 - 93.0 | 67.86 | 64.5 - 71.1 | 2.69 | 0.20 |
| ≤7.23 | 87.65 | 78.5 - 93.9 | 67.61 | 64.2 - 70.9 | 2.71 | 0.18 |
| ≤7.41 | 87.65 | 78.5 - 93.9 | 64.40 | 60.9 - 67.8 | 2.46 | 0.19 |
| ≤7.42 | 88.89 | 80.0 - 94.8 | 64.40 | 60.9 - 67.8 | 2.50 | 0.17 |
| ≤8.75 | 88.89 | 80.0 - 94.8 | 48.02 | 44.5 - 51.6 | 1.71 | 0.23 |
| ≤8.76 | 90.12 | 81.5 - 95.6 | 48.02 | 44.5 - 51.6 | 1.73 | 0.21 |
| ≤8.78 | 90.12 | 81.5 - 95.6 | 47.76 | 44.2 - 51.3 | 1.73 | 0.21 |
| ≤8.79 | 91.36 | 83.0 - 96.5 | 47.76 | 44.2 - 51.3 | 1.75 | 0.18 |
| ≤9.02 | 91.36 | 83.0 - 96.5 | 45.07 | 41.5 - 48.6 | 1.66 | 0.19 |
| ≤9.03 | 92.59 | 84.6 - 97.2 | 45.07 | 41.5 - 48.6 | 1.69 | 0.16 |
| ≤9.79 | 92.59 | 84.6 - 97.2 | 37.90 | 34.5 - 41.4 | 1.49 | 0.20 |
| ≤9.82 | 93.83 | 86.2 - 98.0 | 37.64 | 34.2 - 41.1 | 1.50 | 0.16 |
| ≤10.24 | 93.83 | 86.2 - 98.0 | 34.70 | 31.4 - 38.2 | 1.44 | 0.18 |
| ≤10.25 | 95.06 | 87.8 - 98.6 | 34.70 | 31.4 - 38.2 | 1.46 | 0.14 |
| ≤10.37 | 95.06 | 87.8 - 98.6 | 33.29 | 30.0 - 36.7 | 1.43 | 0.15 |
| ≤10.39 | 96.30 | 89.6 - 99.2 | 33.29 | 30.0 - 36.7 | 1.44 | 0.11 |
| ≤10.47 | 96.30 | 89.6 - 99.2 | 31.75 | 28.5 - 35.1 | 1.41 | 0.12 |
| ≤10.51 | 97.53 | 91.4 - 99.7 | 31.75 | 28.5 - 35.1 | 1.43 | 0.078 |
| ≤11.72 | 97.53 | 91.4 - 99.7 | 20.61 | 17.8 - 23.6 | 1.23 | 0.12 |
| ≤11.73 | 98.77 | 93.3 - 100.0 | 20.61 | 17.8 - 23.6 | 1.24 | 0.060 |
| ≤12.85 | 98.77 | 93.3 - 100.0 | 12.80 | 10.5 - 15.4 | 1.13 | 0.096 |
| ≤12.89 | 100.00 | 95.5 - 100.0 | 12.80 | 10.5 - 15.4 | 1.15 | 0.00 |
| ≤20.77 | 100.00 | 95.5 - 100.0 | 0.00 | 0.0 - 0.5 | 1.00 |  |

**Table S2:** ROC-SPISE coordinates for males

| Criterion | Sensitivity | 95% CI | Specificity | 95% CI | +LR | -LR |
| --- | --- | --- | --- | --- | --- | --- |
| <2.03 | 0.00 | 0.0 - 6.6 | 100.00 | 99.1 - 100.0 |  | 1.00 |
| ≤3.24 | 12.96 | 5.4 - 24.9 | 100.00 | 99.1 - 100.0 |  | 0.87 |
| ≤3.4 | 12.96 | 5.4 - 24.9 | 99.50 | 98.2 - 99.9 | 25.80 | 0.87 |
| ≤3.71 | 27.78 | 16.5 - 41.6 | 99.50 | 98.2 - 99.9 | 55.28 | 0.73 |
| ≤3.72 | 27.78 | 16.5 - 41.6 | 98.99 | 97.4 - 99.7 | 27.64 | 0.73 |
| ≤3.92 | 33.33 | 21.1 - 47.5 | 98.99 | 97.4 - 99.7 | 33.17 | 0.67 |
| ≤4 | 33.33 | 21.1 - 47.5 | 98.49 | 96.7 - 99.4 | 22.11 | 0.68 |
| ≤4.18 | 37.04 | 24.3 - 51.3 | 98.49 | 96.7 - 99.4 | 24.57 | 0.64 |
| ≤4.21 | 38.89 | 25.9 - 53.1 | 97.99 | 96.1 - 99.1 | 19.35 | 0.62 |
| ≤4.36 | 38.89 | 25.9 - 53.1 | 97.49 | 95.4 - 98.8 | 15.48 | 0.63 |
| ≤4.39 | 40.74 | 27.6 - 55.0 | 97.49 | 95.4 - 98.8 | 16.21 | 0.61 |
| ≤4.42 | 40.74 | 27.6 - 55.0 | 96.23 | 93.9 - 97.9 | 10.81 | 0.62 |
| ≤4.46 | 42.59 | 29.2 - 56.8 | 96.23 | 93.9 - 97.9 | 11.30 | 0.60 |
| ≤4.5 | 42.59 | 29.2 - 56.8 | 95.48 | 92.9 - 97.3 | 9.42 | 0.60 |
| ≤4.62 | 48.15 | 34.3 - 62.2 | 95.48 | 92.9 - 97.3 | 10.65 | 0.54 |
| ≤4.68 | 48.15 | 34.3 - 62.2 | 94.22 | 91.5 - 96.3 | 8.33 | 0.55 |
| ≤4.69 | 50.00 | 36.1 - 63.9 | 94.22 | 91.5 - 96.3 | 8.65 | 0.53 |
| ≤4.73 | 50.00 | 36.1 - 63.9 | 93.47 | 90.6 - 95.7 | 7.65 | 0.53 |
| ≤4.74 | 51.85 | 37.8 - 65.7 | 93.47 | 90.6 - 95.7 | 7.94 | 0.52 |
| ≤4.78 | 51.85 | 37.8 - 65.7 | 93.22 | 90.3 - 95.5 | 7.64 | 0.52 |
| ≤4.83 | 53.70 | 39.6 - 67.4 | 92.96 | 90.0 - 95.3 | 7.63 | 0.50 |
| ≤4.87 | 53.70 | 39.6 - 67.4 | 91.96 | 88.8 - 94.4 | 6.68 | 0.50 |
| ≤5.03 | 55.56 | 41.4 - 69.1 | 91.96 | 88.8 - 94.4 | 6.91 | 0.48 |
| ≤5.11 | 55.56 | 41.4 - 69.1 | 91.46 | 88.3 - 94.0 | 6.50 | 0.49 |
| ≤5.14 | 57.41 | 43.2 - 70.8 | 91.46 | 88.3 - 94.0 | 6.72 | 0.47 |
| ≤5.33 | 57.41 | 43.2 - 70.8 | 89.70 | 86.3 - 92.5 | 5.57 | 0.47 |
| ≤5.36 | 59.26 | 45.0 - 72.4 | 89.45 | 86.0 - 92.3 | 5.62 | 0.46 |
| ≤5.37 | 59.26 | 45.0 - 72.4 | 89.20 | 85.7 - 92.1 | 5.48 | 0.46 |
| ≤5.45 | 62.96 | 48.7 - 75.7 | 89.20 | 85.7 - 92.1 | 5.83 | 0.42 |
| ≤5.52 | 62.96 | 48.7 - 75.7 | 88.94 | 85.4 - 91.9 | 5.70 | 0.42 |
| ≤5.53 | 64.81 | 50.6 - 77.3 | 88.69 | 85.2 - 91.6 | 5.73 | 0.40 |
| ≤5.8 | 64.81 | 50.6 - 77.3 | 86.93 | 83.2 - 90.1 | 4.96 | 0.40 |
| ≤5.82 | 66.67 | 52.5 - 78.9 | 86.93 | 83.2 - 90.1 | 5.10 | 0.38 |
| ≤5.9 | 66.67 | 52.5 - 78.9 | 85.93 | 82.1 - 89.2 | 4.74 | 0.39 |
| ≤5.91 | 68.52 | 54.4 - 80.5 | 85.93 | 82.1 - 89.2 | 4.87 | 0.37 |
| ≤5.94 | 68.52 | 54.4 - 80.5 | 85.43 | 81.6 - 88.7 | 4.70 | 0.37 |
| ≤5.98 | 70.37 | 56.4 - 82.0 | 85.43 | 81.6 - 88.7 | 4.83 | 0.35 |
| ≤6.13 | 70.37 | 56.4 - 82.0 | 83.92 | 79.9 - 87.4 | 4.38 | 0.35 |
| **≤6.14** | **72.22** | **58.4 - 83.5** | **83.92** | **79.9 - 87.4** | **4.49** | **0.33** |
| ≤6.35 | 72.22 | 58.4 - 83.5 | 81.41 | 77.2 - 85.1 | 3.88 | 0.34 |
| ≤6.37 | 74.07 | 60.3 - 85.0 | 81.41 | 77.2 - 85.1 | 3.98 | 0.32 |
| ≤6.61 | 74.07 | 60.3 - 85.0 | 79.15 | 74.8 - 83.0 | 3.55 | 0.33 |
| ≤6.62 | 75.93 | 62.4 - 86.5 | 79.15 | 74.8 - 83.0 | 3.64 | 0.30 |
| ≤6.9 | 75.93 | 62.4 - 86.5 | 74.87 | 70.3 - 79.1 | 3.02 | 0.32 |
| ≤6.93 | 77.78 | 64.4 - 88.0 | 74.87 | 70.3 - 79.1 | 3.10 | 0.30 |
| ≤7.08 | 77.78 | 64.4 - 88.0 | 73.12 | 68.5 - 77.4 | 2.89 | 0.30 |
| ≤7.09 | 79.63 | 66.5 - 89.4 | 73.12 | 68.5 - 77.4 | 2.96 | 0.28 |
| ≤7.1 | 81.48 | 68.6 - 90.7 | 72.86 | 68.2 - 77.2 | 3.00 | 0.25 |
| ≤7.41 | 81.48 | 68.6 - 90.7 | 68.34 | 63.5 - 72.9 | 2.57 | 0.27 |
| ≤7.42 | 83.33 | 70.7 - 92.1 | 68.34 | 63.5 - 72.9 | 2.63 | 0.24 |
| ≤8.75 | 83.33 | 70.7 - 92.1 | 54.52 | 49.5 - 59.5 | 1.83 | 0.31 |
| ≤8.76 | 85.19 | 72.9 - 93.4 | 54.52 | 49.5 - 59.5 | 1.87 | 0.27 |
| ≤8.78 | 85.19 | 72.9 - 93.4 | 54.27 | 49.2 - 59.2 | 1.86 | 0.27 |
| ≤8.79 | 87.04 | 75.1 - 94.6 | 54.27 | 49.2 - 59.2 | 1.90 | 0.24 |
| ≤9.02 | 87.04 | 75.1 - 94.6 | 51.51 | 46.5 - 56.5 | 1.79 | 0.25 |
| ≤9.03 | 88.89 | 77.4 - 95.8 | 51.51 | 46.5 - 56.5 | 1.83 | 0.22 |
| ≤9.76 | 88.89 | 77.4 - 95.8 | 43.97 | 39.0 - 49.0 | 1.59 | 0.25 |
| ≤9.82 | 90.74 | 79.7 - 96.9 | 43.47 | 38.5 - 48.5 | 1.61 | 0.21 |
| ≤10.14 | 90.74 | 79.7 - 96.9 | 40.45 | 35.6 - 45.5 | 1.52 | 0.23 |
| ≤10.25 | 92.59 | 82.1 - 97.9 | 40.45 | 35.6 - 45.5 | 1.55 | 0.18 |
| ≤10.37 | 92.59 | 82.1 - 97.9 | 38.19 | 33.4 - 43.2 | 1.50 | 0.19 |
| ≤10.39 | 94.44 | 84.6 - 98.8 | 38.19 | 33.4 - 43.2 | 1.53 | 0.15 |
| ≤10.45 | 94.44 | 84.6 - 98.8 | 36.18 | 31.5 - 41.1 | 1.48 | 0.15 |
| ≤10.51 | 96.30 | 87.3 - 99.5 | 36.18 | 31.5 - 41.1 | 1.51 | 0.10 |
| ≤11.72 | 96.30 | 87.3 - 99.5 | 24.62 | 20.5 - 29.2 | 1.28 | 0.15 |
| ≤11.73 | 98.15 | 90.1 - 100.0 | 24.62 | 20.5 - 29.2 | 1.30 | 0.075 |
| ≤12.85 | 98.15 | 90.1 - 100.0 | 15.33 | 11.9 - 19.2 | 1.16 | 0.12 |
| ≤12.89 | 100.00 | 93.4 - 100.0 | 15.33 | 11.9 - 19.2 | 1.18 | 0.00 |
| ≤20.77 | 100.00 | 93.4 - 100.0 | 0.00 | 0.0 - 0.9 | 1.00 |  |

Table S3: ROC-SPISE coordinates for females

| Criterion | Sensitivity | 95% CI | Specificity | 95% CI | +LR | -LR |
| --- | --- | --- | --- | --- | --- | --- |
| <2.15 | 0.00 | 0.0 - 12.8 | 100.00 | 99.0 - 100.0 |  | 1.00 |
| ≤2.15 | 3.70 | 0.09 - 19.0 | 100.00 | 99.0 - 100.0 |  | 0.96 |
| ≤2.44 | 3.70 | 0.09 - 19.0 | 99.74 | 98.6 - 100.0 | 14.19 | 0.97 |
| ≤2.95 | 18.52 | 6.3 - 38.1 | 99.74 | 98.6 - 100.0 | 70.93 | 0.82 |
| ≤2.97 | 18.52 | 6.3 - 38.1 | 99.48 | 98.1 - 99.9 | 35.46 | 0.82 |
| ≤3.49 | 29.63 | 13.8 - 50.2 | 99.48 | 98.1 - 99.9 | 56.74 | 0.71 |
| ≤3.65 | 29.63 | 13.8 - 50.2 | 98.69 | 97.0 - 99.6 | 22.70 | 0.71 |
| ≤3.69 | 33.33 | 16.5 - 54.0 | 98.43 | 96.6 - 99.4 | 21.28 | 0.68 |
| ≤3.74 | 33.33 | 16.5 - 54.0 | 98.17 | 96.3 - 99.3 | 18.24 | 0.68 |
| ≤3.76 | 37.04 | 19.4 - 57.6 | 97.91 | 95.9 - 99.1 | 17.73 | 0.64 |
| ≤3.81 | 37.04 | 19.4 - 57.6 | 97.65 | 95.6 - 98.9 | 15.76 | 0.64 |
| ≤3.85 | 48.15 | 28.7 - 68.1 | 97.65 | 95.6 - 98.9 | 20.49 | 0.53 |
| ≤4.18 | 48.15 | 28.7 - 68.1 | 96.34 | 93.9 - 98.0 | 13.17 | 0.54 |
| ≤4.19 | 51.85 | 31.9 - 71.3 | 96.08 | 93.6 - 97.8 | 13.24 | 0.50 |
| ≤4.56 | 51.85 | 31.9 - 71.3 | 92.69 | 89.6 - 95.1 | 7.09 | 0.52 |
| ≤4.57 | 59.26 | 38.8 - 77.6 | 92.43 | 89.3 - 94.9 | 7.83 | 0.44 |
| ≤4.89 | 59.26 | 38.8 - 77.6 | 90.60 | 87.2 - 93.3 | 6.30 | 0.45 |
| ≤4.9 | 62.96 | 42.4 - 80.6 | 90.60 | 87.2 - 93.3 | 6.70 | 0.41 |
| ≤5.22 | 62.96 | 42.4 - 80.6 | 85.64 | 81.7 - 89.0 | 4.38 | 0.43 |
| ≤5.26 | 66.67 | 46.0 - 83.5 | 85.64 | 81.7 - 89.0 | 4.64 | 0.39 |
| ≤5.28 | 66.67 | 46.0 - 83.5 | 85.12 | 81.2 - 88.5 | 4.48 | 0.39 |
| ≤5.31 | 70.37 | 49.8 - 86.2 | 85.12 | 81.2 - 88.5 | 4.73 | 0.35 |
| ≤5.51 | 70.37 | 49.8 - 86.2 | 83.03 | 78.9 - 86.7 | 4.15 | 0.36 |
| ≤5.52 | 74.07 | 53.7 - 88.9 | 83.03 | 78.9 - 86.7 | 4.36 | 0.31 |
| ≤5.54 | 74.07 | 53.7 - 88.9 | 82.77 | 78.6 - 86.4 | 4.30 | 0.31 |
| ≤5.56 | 77.78 | 57.7 - 91.4 | 82.77 | 78.6 - 86.4 | 4.51 | 0.27 |
| ≤5.65 | 77.78 | 57.7 - 91.4 | 81.20 | 76.9 - 85.0 | 4.14 | 0.27 |
| ≤5.69 | 81.48 | 61.9 - 93.7 | 80.94 | 76.6 - 84.8 | 4.27 | 0.23 |
| ≤6.23 | 81.48 | 61.9 - 93.7 | 75.20 | 70.6 - 79.4 | 3.28 | 0.25 |
| ≤6.24 | 88.89 | 70.8 - 97.6 | 75.20 | 70.6 - 79.4 | 3.58 | 0.15 |
| ≤6.31 | 88.89 | 70.8 - 97.6 | 74.41 | 69.7 - 78.7 | 3.47 | 0.15 |
| ≤6.33 | 92.59 | 75.7 - 99.1 | 74.41 | 69.7 - 78.7 | 3.62 | 0.100 |
| ≤6.42 | 92.59 | 75.7 - 99.1 | 73.37 | 68.6 - 77.7 | 3.48 | 0.10 |
| **≤6.46** | **96.30** | **81.0 - 99.9** | **73.37** | **68.6 - 77.7** | **3.62** | **0.050** |
| ≤7.19 | 96.30 | 81.0 - 99.9 | 64.23 | 59.2 - 69.0 | 2.69 | 0.058 |
| ≤7.23 | 100.00 | 87.2 - 100.0 | 63.97 | 58.9 - 68.8 | 2.78 | 0.00 |
| ≤20.67 | 100.00 | 87.2 - 100.0 | 0.00 | 0.0 - 1.0 | 1.00 |  |
